# A New Amalgam for Filling Teeth

**Published:** 1849-04

**Authors:** 


*A New Amalgam for Filling Teeth.*—Mr. Evans, late of Lancaster, Pa., but now an associate of Dr. Brewster of Paris, recommends in a late number of the London Lancet, an amalgam which he conceives to be free from the objections possessed by all other combinations of mercury. The error into which Mr. E. has evidently fallen, in relation to this matter, is thus pointed out in a subsequent number of the Lancet, by Mr. J. L. Levison, an eminent English dentist.—*Balt. Ed.*

